# High prevalence of low back pain across the lifespan in Brazilian elderly: Temporal trends and multifactorial associations in Barra Mansa

**DOI:** 10.1016/j.clinsp.2025.100747

**Published:** 2025-08-05

**Authors:** Priscila de Oliveira Januário, Ingred Merllin Batista de Souza, Ariela Torres Cruz, Mateus Dias Antunes, Mara Maria Lisboa Santana Pinheiro, Anice de Campos Pássaro, Deizyane dos Reis Galhardo, João Simão de Mello Neto, Bianca Callegari, Amélia Pasqual Marques

**Affiliations:** a Department of Physiotherapy, Speech Therapy and Occupational Therapy, Faculdade de Medicina da Universidade de São Paulo (FMUSP), São Paulo, SP, Brazil; b Department of Physiotherapy, Centro Universitário Barra Mansa (UBM), Barra Mansa, RJ, Brazil; c Clinical and Experimental Research Unit of the Urogenital System, Instituto de Ciências da Saúde da Universidade Federal do Pará (UFPA), Belém, PA, Brazil; d Laboratory of Human Motricity Sciences, Universidade Federal do Pará (UFPA), Belém, PA, Brazil

**Keywords:** Prevalence, Low back pain, Aged, Risk factors, Epidemiology

## Abstract

**Background:**

This Cross-sectional study with 516 elderly individuals aimed to measure the prevalence of Low Back Pain (LBP) at different periods and identify associated factors in the elderly population from Barra Mansa, Rio de Janeiro.

**Methods:**

A questionnaire on the prevalence of LBP was applied, covering three periods: at the time of the interview, in the last year, and at some point in life, with sociodemographic, clinical, behavioral, and occupational variables in addition, the Numerical Pain Scale and the Roland Morris questionnaire. The significance level established was 5 %.

**Results:**

The prevalence of LBP was high at the time of the interview, 52.1 % (95 % CI 0.467‒0.575), 93 % in the last year (95 % CI 0.899‒0.954), and 77.4 % at some point in life (95 % CI 0.710‒0.800). The intensity of pain and functional incapacity were considered moderate. The LBP was associated with lower body mass index, female sex, high blood pressure, greater functional disability and perception of regular health. More active elderly people had less LBP, while the white race had a higher prevalence, and emotional problems had impacted severely at the time of the interview. Diabetes mellitus and being widowed were associated with a greater prevalence of LBP at some point in life. Greater functional disability worsened LBP at the time of the interview, while lower disability increased the prevalence of LBP at some point in life. Greater pain intensity was associated to higher LBP in the last year.

**Conclusion:**

The findings indicate a high prevalence of LBP in different periods associated with sociodemographic, clinical and behavioral factors.

## Highlights

• High temporal prevalence: 52.1 % (current), 93 % (past year), 77.4 % (lifetime) LBP in elderly Brazilians.
• Key risk factors: Female sex, hypertension, lower BMI, and functional disability drive LBP burden.
• Protective effect: Very active elderly had 74 % lower odds of current LBP (*p* < 0.05).
• Novel associations: Widowhood and diabetes significantly increased lifetime LBP risk.
• Clinical implication: Community-based exercise programs may reduce disability and healthcare costs.

## Introduction

The Low Back Pain (LBP) is defined as pain located below the costal margin and above the inferior gluteal fold, with or without pain that may radiate to the lower limbs. The majority (90 %) of LBP presentations are nonspecific, meaning that the etiology is unknown, and the diagnosis is made based on the exclusion of a specific pathology. It can also be classified according to the duration of symptoms as acute LBP (less than six weeks), subacute (more than six weeks and less than three months), and chronic (three months)^1^^,^^2^

The estimated global LBP is 70 % to 80 % throughout life, affecting 569 million people, with a specific prevalence age-standardized of 6, 972.5 per 100,000 inhabitants [1.3] A systematic review using data from the international literature on the prevalence of LBP in the elderly population (in developing or developed countries) indicates a high prevalence of LBP among the elderly, ranging from 21.7 % to 75 %^4^ In the first national meta-analysis that investigated the prevalence of LBP among elderly people in Brazil, Leopoldino et al. (2016)^5^ indicated moderate-quality evidence, and the punctual prevalence of LBP in elderly Brazilians was 25.0 % (95 % CI 18.0 %–32.0 %).

The prevalence of LBP increases with age, reaching 21,762.12 cases among people aged 70-years and over. The state with the highest prevalence rate of LBP regarding age/sex is Rio Grande do Sul, and with the lowest rates is Amapá. The states of the south and southeast tend to have a higher prevalence of LBP than the states of the north and northeast, and the difference between the states with the highest and lowest prevalence is 32 %^3^

Although only 10 % to 20 % of individuals with LBP progress to the chronic stage, this group faces intense pain, functional disability, and limitations in daily activities, in addition to biophysical, psychological and social impairments, making the elderly population more dependent, vulnerable, and with reduced quality of life^2^^,^^4^

The costs associated with lost productivity are likely to be substantial, as the overall prevalence of chronic LBP in countries with low- and middle-income is estimated to be around 52 % of workers. >80 % of the total costs related to LBP are due to indirect costs such as lost productivity and disability payments. The lack of adherence to the LBP treatment guidelines may be associated with the direct increased costs of healthcare^6^, ^7^, ^8^, ^9^

The increase in life expectancy in a country like Brazil, with continental dimensions and socioeconomic inequalities, can lead to significant limitations for the elderly population. Between 2013 and 2018, the southeast region of the country had the highest financial expenditure for the treatment of LBP^10^

This research is part of studies conducted in other Brazilian cities, and there is a lack of prevalence data from the Southeast region, which is one of the most economically active regions in Brazil. Since other studies have been conducted in large capitals, it is important to conduct research in cities in the interior, such as Barra Mansa. Historically, the city has shown lower gross domestic product growth with the arrival of industries set up in neighboring cities and currently, the service sector is the most relevant in the municipality, which makes it a low performer in generating wealth in this segment, compared to neighboring cities, the state of Rio de Janeiro and the Brazilian performance^11^ In addition, this study measures temporal variations in low back pain, which is rare in the existing literature with the elderly population.

Although LBP has been identified as a major health problem, its prevalence is still little known in the elderly population. Understanding the sociodemographic, behavioral, occupational, and general health factors associated with the prevalence of LBP in the elderly population is essential, as they are important sources of information for the development of public policies that prioritize health, in addition to supporting preventive measures and/or therapeutic interventions^12^ Therefore, this study aimed to measure the prevalence of pain at different periods and verify associated factors in the elderly population in the city of Barra Mansa, Rio de Janeiro.

## Materials and methods

### *Study design*

This observational cross-sectional study followed the Strengthening the Reporting of Observational Studies in Epidemiology guidelines (STROBE)^13^ and it was approved by the Research and Ethics Committee of the Medicine faculty at the University of São Paulo (permission nº: 1.581.420), and the research participants signed the Free and Informed Consent Form (FICF). The study included 516 elderly people aged 60-years or older, of both sexes, with or without LBP, living in the city of Barra Mansa, Rio de Janeiro.

### *Participants and sample size*

The inclusion criteria were: Elderly people aged 60-years or older; both sexes, living in the urban perimeter and eastern region of the city of Barra Mansa-RJ. They excluded: elderly people with previous spinal surgery, undergoing physiotherapy treatment for LBP, restricted to a wheelchair or with a walking aid and elderly people living in the rural region of Barra Mansa.

The sample calculation was based on the systematic review by Hoy et al., (2012)^14^ The parameters used considered the general elderly population from the city of Barra Mansa (*n* = 21,655) according to the census from the Brazilian Institute of Geography and Statistics (IBGE-2010),^15^ since the study began in 2019. The adjusted mean prevalence in the last month was 23.2 % (*p* = 0.232), precision of 4 % (*p* = 0.04), confidence interval of 95 % (*z* = 1.96), allowing a sample loss of 20 % due to participant refusals and incomplete questionnaires. The total study sample is 513 people.

### *Procedures*

Data collection was carried out from June 2019 to December 2023, with elderly people recruited through an on-site visit, following safety protocols due to COVID-19. The Collection took place in a single approach, using the Google Forms platform to apply the questionnaires. The research team was made up of six scientific initiation students, two physiotherapists, and two master teachers, all previously trained by the main researcher. The training included explanations about the approaches, collection locations, filling out the questionnaires, signing the FICF, and applying the questionnaires. The interviews, lasting fifteen minutes, took place in Basic Health Units, the Elderly Reference Center, Nove de Abril Polyclinic, and the Integrated Health Center of Barra Mansa University Center (UBM), with elderly people who participated in activities such as painting and embroidery, and their attendants. The questionnaires were completed digitally through links sent via WhatsApp by the researchers themselves, with explanations provided to the elderly when necessary. The participants were asked about the presence of pain at the moment or in the last three months, which determined the questionnaires to be applied. The participants were informed about the voluntary nature of participation and the assurance of data confidentiality.

## Evaluation

### *Primary outcome variables*

#### Prevalence of low back pain

Regarding to the prevalence of LBP, the volunteers were questioned at three different times: at the time of the interview, in the last year, and at some point in their lives, with information being collected on the frequency, intensity, duration, and irradiation of LBP, according to the LBP standardizing consensus in prevalence studies^14^ For this study, there was considered a LBP episode of LBP as “any pain between the last rib and the bottom of the buttocks, lasting more than twenty-four hours, preceded by thirty days without pain”^16^ In order to facilitate the exact identification of the lower back, an illustrative figure of the human body was used, specifying the lower back with dotted lines^17^

#### Pain intensity

For the subjective evaluation of the intensity of pain reported by the participant, the Pain Numerical Rating Scale (Pain NRS) was used^18^ This is an eleven-point scale, ranging from zero to ten, with zero being characterized as the absence of pain and ten as the most unbearable level of pain felt by the volunteer. The elderly were asked about the presence of pain specifically located in the lower back and, later, using the scale, the number corresponding to the intensity of the pain was identified^16^

#### Functional disability

The Roland-Morris questionnaire was used to assess functional disability, consisting of 24 items that describe everyday situations in which people have difficulty performing some tasks due to LBP. Participants were instructed to report the items that actually described them on the day of the questionnaire was made. The greater the number of alternatives filled in, the greater the disability, and it is being more recommended for a population with low functional disability^19^, ^20^, ^21^ The instrument is easy and quick to apply, with an average response time of five minutes^22^ The score is obtained by adding the items, which range from zero (no disability) to twenty-four (severe disability). Scores above fourteen indicate physical disability, and a clinically relevant difference corresponds to five points^23^

### *Other variables*

#### Sociodemographic variables

Regarding sociodemographic variables, initially, a questionnaire containing personal data of the participants was applied. In the sociodemographic characteristics, the following were recorded: sex, age, marital status, self-declared race/color according to the Brazilian Institute of Geography and Statistics (IBGE), besides it was taken into account the socioeconomic level, education (years of study), and the individual income. Education was stratified into low (complete or incomplete primary and secondary education) and high (university level), while the income was collected based on the Brazilian Economic Classification Criteria, established by the Brazilian Association of Research Companies,^24^ divided into high (A1+A2+B1), medium (B2+*C*), and low (*D* + *E*).

#### Clinical variables

Clinical characteristics included anthropometric variables: weight and height, referred to for the subsequent calculation of Body Mass Index (BMI), stratified according to age group and recommended by the Ministry of Health^25^ Besides that, the participants were asked about alcohol and tobacco consumption, with the categories: non-smokers (never smoked), former smokers (who gave up the habit more than a year ago) and current smokers (who consume any number of cigarettes per day), besides the self-reporting of illnesses^26^.

#### Behavioral variables

Regarding to behavioral variables, the level of physical activity was evaluated using the International Physical Activity Questionnaire (IPAQ) short version, which takes into account the frequency, duration and intensity of the activity, stratifying the individual into: sedentary (no physical activity for ten minutes continuous), insufficiently active (light activities lasting ten minutes on five days a week), active (moderate activities lasting more than twenty minutes three to five days a week) and very active (vigorous activities lasting more than thirty minutes and for more than five days a week)^27^

#### Occupational variables

For occupational variables, the information referred to the work situation, considering those who had a job and those who did not. In addition, data were collected on the length of employment in years, the frequency of exposure (sitting, standing, vibrations/trepidations, squatting, lying down, kneeling, carrying weight and repetitive movements) and job satisfaction, considering as satisfied those who felt good performing the profession and as dissatisfied those who did not feel fulfilled, according to the Brazilian Classification of Occupations^28^ For this study, occupations were stratified into categories: A) Armed Forces, Police and Military Firefighters; B) Senior members of government, directors of public interest organizations, companies and managers; C) Professionals in science and arts; D) High school technicians; E) Agricultural, forestry, hunting and fishing workers; F) Service workers, salespeople in stores and markets; G) Administrative service workers; H) Workers in the production of industrial goods and services; I) Maintenance and repair workers.

### *Statistical analysis*

The descriptive data were presented as absolute and relative frequencies, mean and standard deviation (parametric data), and median (non-parametric data). To verify normality, the data were submitted to the Kolmogorov-Smirnov test. To determine whether there was a statistically significant difference between the variables, the Chi-Square (χ²), *t*-Student (*t*) and *U* Mann Whitney (*U*) tests were used. The binary logistic regression analysis was used to determine the relevant factors in the prevalence of LBP. Initially, the multicollinearity of the variables was checked using the Variance Inflation Factor (VIF < 10) and tolerance > 0.2, which were excluded from the model. Following an univariate analysis was then carried out, considering a p value < 0.25 to include the variables in the multivariate analysis. The statistical significance level was defined as *p* < 0.05^29^ Odds Ratio (OR) with 95 % Confidence Intervals (95 % CI) were used to quantify the degree of association. The statistical tests were carried out using SPSS Statistics Program ‒ IBM®.

## Results

The sample was made up of 516 elderly people with a mean age of (70.06 ± 6.71 years) of whom 64.9 % (95 % CI: 60.06‒68.9) had LBP. When associated to sociodemographic, clinical and behavioral characteristics with the presence of LBP, it the authors found that a lower BMI (*p* = 0.004), being female (*p* < 0.001), having Systemic Arterial Hypertension (SAH) (*p* = 0.003), greater functional incapacity (*p* = 0.025) and regular health perception (*p* < 0.001) showed a significant association. The mean functional disability was 11.93 ± 6.08, with the highest Roland Morris score ≥14 points in 42.2 % of the 227 elderly people, considered between moderate and severe.

**Fig. 1:**
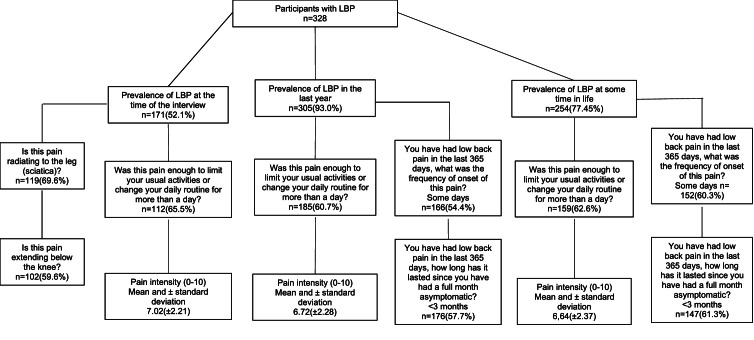
Prevalence and intensity of low back pain in the elderly.

The authors found a high prevalence of LBP at the time of the interview, in the last year, and at some point in life. It has been reported irradiated LBP to the lower limbs (leg 95 % CI 0.632‒0.736/knee 0.527‒0.639), with a frequency of a few days (95 % CI 0.237‒0.339), duration of <3-months (95 % CI: 0.237‒0.339/0.102‒0.181) and sufficient to limit usual activities (95 % CI 0.553‒0.662). The pain intensity was moderate (95 % CI 6.47‒6.98) in 328 elderly people (Fig. 1).

### *Prevalence of low back pain at the time of interview*

The anthropometric variables: weight, height, and BMI, did not pass the multicollinearity test and were excluded from the model. During the univariate regression, the variables selected for the multivariate analysis (*p* < 0.25) were spatial distribution and race (Table 1). The results indicate that people who live in the West Zone have lower LBP at the time of the interview than those who live in the East Zone. Furthermore, white people had more LBP at the time of the interview than people of Black Race/Color (*p* < 0.05) (Table 1). All variables related to lifestyle habits passed the multicollinearity test. The variables physical activity, health perception, and emotional level were included in the multivariate pain at the time of the interview, analysis (*p* < 0.25) (Table 2). The authors observed that the very active elderly people had less LBP at the time of the interview than the sedentary ones (*p* < 0.05) (Table 2). The very active elderly people had less LBP at the time of the interview than the sedentary ones (*p* < 0.05) (Table 2). People with fair, good, or excellent health perception had less pain than those with poor perception (*p* < 0.05). When asked about how physical health or emotional problems impacted social activities, the category slightly, moderately, or severely shows more association than not at all (Table 2). The exposure time of the previous occupation was excluded from the model due to collinearity. The occupational health and functionality variables associated with low back pain at the time of the interview included in the multivariate regression were satisfaction with the previous occupation, current occupation, pain scale, and functional capacity (Table 3). The authors observed that satisfaction with previous occupation, current occupation, pain scale, and functional disability are related. The greater the functional disability, the worse the LBP at the time of the interview (Table 3).

**Table 1:** Sociodemographic characteristics associated with low back pain at the time of the interview of the 328 elderly people.

	OR^a^	95% CI^a^	p-value^a^	OR^b^	95% CI^b^	p-value^b^^b^
Spatial distribution (ref. East Zone)	0.706	0.541, 0.923	0.011^c^	0.699	0.534, 0.915	0.009^d^
North	0.563	0.201, 1.572	0.272	0.542	0.185, 1.589	0.264
West	0.327	0.116, 0.917	0.034^c^	0.309	0.105, 0.913	0.034^d^
South	0.331	0.112, 0.974	0.045^c^	0.328	0.106, 1.019	0.054
Sex (ref. Female)	0.897	0.542, 1.485	0.673	‒	‒	‒
Male	1.114	0.674, 1.844	0.673	‒	‒	‒
Age (continuous)	0.981	0.947, 1.017	0.300	‒	‒	‒
Race (ref. Black)	0.854	0.711, 1.026	0.091^c^	0.849	0.705, 1.022	0.084
Yellow	0.500	0.050, 5.022	0.556	0.643	0.062, 6.674	0.712
White	1.967	1.088, 3.555	0.025^c^	1.949	1.053, 3.606	0.034^d^
Indígenous	0.000	0.000	1.000	0.000	0.000	1.000
Mixed race	1.676	0.934, 3.011	0.084^c^	1.645	0.896,3.020	0.109

Classes A‒E, Economic Classification of Brazil established by the Brazilian Association of Research Companies; Ref, Reference;.

a OR, Odds Ratio;

b aOR, Adjusted Odds Ratio; CI, Confidence Interval.

c p-value < 0.25;

d p-value < 0.05.

**Table 2:** Clinical and behavioral characteristics associated with low back pain at the time of the interview of the 328 elderly.

	OR^a^	95% CI^a^	p-value^a^	OR^b^	95% CI^b^	p-value^b^
Physical activity (ref. Sedentary)	0.895	0.708, 1.131	0.351	0.934	0.736, 1.186	0.602
Insufficiently active	4.667	1.298, 16.778	0.018^c^	1.600	0.737, 3.477	0.236
Active	6.533	1.592, 26.815	0.009^c^	1.455	0.779, 2.719	0.239
Very active	5.744	1.489, 22.157	0.011^c^	0.259	0.070, 0.959	0.052
Health Perception (ref. Poor)	0.707	0.542, 0.923	0.011^c^	0.715	0.546, 0.936	0.028^d^
Regular	18.200	1.979, 167.337	0.010^c^	0.331	0.143, 0.765	0.014^d^
Good	6.781	0.811, 56.733	0.077^c^	0.319	0.136., 0.748	0.006^d^
Very Good	5.914	0.703, 49.740	0.102^c^	0.437	0.133, 1.430	0.165
Excellent	8.556	0.881, 83.057	0.064^c^	0.051	0.005, 0.489	0.007^d^
Emotional Level** (Ref. Not at all)	1.266	1.063, 1.508	0.008^c^			0.017^d^
Slightly	1.126	0.538, 2.356	0.753	1.027	0.471, 2.239	0.010^d^
Moderately	2.111	0.971, 4.592	0.059	1.960	0.869, 4.423	0.009^d^
Quite	2.137	1.074, 4.255	0.031^c^	1.969	0.955, 4.063	0.171
Severely	1.990	0.736, 5.383	0.175	1.793	0.632, 5.084	0.010^d^

Ref, Reference;

a OR, Odds Ratio;

b aOR, Adjusted Odds Ratio; CI, Confidence Interval.

c p-value < 0.25;

d p-value < 0.05.

**Table 3:** Occupation and functional disability associated with low back pain at the time of the interview of the 328 participants.

	OR^a^	95% CI^a^	p-value^a^	OR^b^	95% CI^b^	p-value^b^
Previous Occupation Satisfaction (ref. Unsatisfied)	0.935	0.628, 1.393	0.743	1.038	0.627, 1.719	0.884
Satisfied	0.464	0.166, 1.297	0.143^c^	0.941	0.249, 3.555	0.928
Very Satisfied	0.539	0.188, 1.543	0.250	1.069	0.274, 4.167	0.923
Current Occupation (ref. No Occupation)	0.966	0.876, 1.065	0.486	0.982	0.868, 1.111	0.774
Category A	‒	‒	‒	‒	‒	‒
Category B	0.975	0.060, 15.774	0.986	19,971,216	0.000	1.000
Category C	2.926	0.579, 14.788	0.194^c^	11,824,857	0.000	0.999
Category D	0.975	0.060, 15.774	0.986	12,386,463	0.000	1.000
Category E	0.000	0.000	1.000	‒	‒	‒
Category F	0.813	0.392, 1.687	0.578	0.785	0.326, 1.889	0.589
Category G	‒	‒	‒	‒	‒	‒
Category H	0.650	0.179, 2.363	0.513	0.721	0.127, 1.4.083	0.712
Category I	‒	‒	‒	‒	‒	‒
Pain Scale (Continuous)	1.147	1.038, 1.267	0.007^c^	1.036	0.901, 1.192	0.620
Functional Disability (Continuous)	1.114	1.058, 1.172	<0.001^c^	1.107	1.043, 1.174	0.001^d^

Categories A‒I, Brazilian Classification of Occupations; Ref, Reference;

a OR, Odds Ratio;

b aOR, Adjusted Odds Ratio; CI, Confidence Interval.

c p-value < 0.25;

d p-value < 0.05.

### Prevalence of low back pain in the last year

The anthropometric variables: weight, height, and BMI, did not pass the multicollinearity test and were excluded from the models. During the univariate regression, the variables selected for the multivariate analysis (*p* < 0.25) were spatial distribution, sex, age, income, health perception, smoking, alcoholism and SAH (Table 4, Table 5). The variables sex, age, income, health perception, smoking, alcoholism, and SAH were not associated with a higher prevalence of LBP in the last year (Table 4, Table 5). The time of exposure to the previous occupation was excluded from the model due to collinearity. In the univariate regression, the variables selected for multivariate analysis (*p* < 0.25) were satisfaction with the previous occupation, current occupation, pain scale, and functional disability (Table 6). On the other hand, the authors found that greater pain intensity and greater functional disability were associated with a higher prevalence of LBP in the last year (*p* < 0.05) (Table 6). In addition, satisfaction with previous occupation and current occupation also demonstrated a significant association with the prevalence of LBP in the last year (*p* < 0.05) (Table 6).

**Table 4:** Sociodemographic characteristics associated with low back pain in the last year of the 328 participants.

	OR^a^	95% CI^a^	p-value^a^	OR^b^	95% CI^b^	p-value^b^
Spatial distribution (ref. East Zone)	1.425	0.856, 2.370	0.173^c^	1.367	0.810, 2.309	0.242
North	0.532	0.064, 4.399	0.558	0.463	0.054, 3.965	0.482
West	0.445	0.054, 3.655	0.451	0.412	0.048, 3.507	0.417
South	85,024,991,73	0.000	0.997	63,569,591.95	0.000	0.997
Sex (Ref. Female)	0.450	0.187, 1.085	0.075^c^	0.447	0.181, 1.099	0.079
Male	0.450	0.187, 1.085	0.075^c^	0.547	0.212, 1.414	0.213
Age (continuous)	0.948	0.890, 1.009	0.095^c^	0.960	0.900, 1.025	0.219
Income (Ref. No income)	0.667	0.411, 1.083	0.102^c^	0.667	0.389, 1.145	0.142
Class A	1.000	0.000	1.000	3.321	0.000	1.000
Class B	1.000	0.000	1.000	2.320	0.000	1.000
Class C	0.000	0.000	0.998	0.000	0.000	0.998
Class D	0.000	0.000	0.998	0.000	0.000	0.998
Class E	0.000	0.000	0.998	0.000	0.000	0.998

Classes A‒E, Economic Classification of Brazil established by the Brazilian Association of Research Companies; Ref, Reference;.

a OR, odds Ratio;

b aOR, Adjusted Odds Ratio; CI, Confidence Interval.

c p-value < 0.25; ^d^ p-value < 0.05.

**Table 5:** Características clínicas e comportamentais associadas à dor lombar no último ano dos 328 participantes.

	OR^a^	95% CI^a^	p-value^a^	OR^b^	95% CI^b^	p-value^b^
Health Perception (ref. Poor)	0.743	0.466, 1.186	0.213^c^	0.678	0.409, 1.122	0.130
Regular	1.176	0.227, 6.095	0.846	1.244	0.236, 6.558	0.797
Good	0.466	0.099, 2.188	0.333	0.419	0.087, 2.025	0.279
Very Good	1.117	0.095, 13.150	0.930	0.996	0.079, 12.513	0.998
Excellent	0.412	0.033, 5.193	0.493	0.329	0.023, 4.631	0.410
Smoking (ref. Smoker)	0.680	0.363, 1.275	0.229^c^	0.670	0.345, 1.301	0.237
Never Smoked	1.600	0.328, 7.803	0.561	1.847	0.351, 9.713	0.469
Ex-smoker	0.819	0.167, 4.022	0.806	0.904	0.177, 4.624	0.904
Alcoholism	0.573	0.223, 1.470	0.247^c^	0.658	0.240, 1.805	0.416
Alcohol Consumption						
(Ref. Once a month or less)	0.974	0.595, 1.594	0.917	‒	‒	‒
Two to four times a month	0.401	0.136, 1.185	0.098	‒	‒	‒
Two to three times a week	0.449	0.093, 2.177	0.320	‒	‒	‒
Four or more times a week	111,668,767.9	0.000	0.999	‒	‒	‒
Systemic Arterial Hypertension	0.514	0.169, 1.565	0.242^c^	0.384	0.119, 1.240	0.109

Ref, Reference;

a OR, Odds Ratio;

b aOR, Adjusted Odds Ratio; CI, Confidence Interval.

c p-value < 0.25; * p-value < 0.05.

**Table 6:** Occupation and functional disability associated with low back pain in the last year of the 328 participants.

	OR^a^	95% CI^a^	p-value^a^	OR^b^	95% CI^b^	p-value^b^
Satisfaction Previous Occupation (Ref. Unsatisfied)	0.484	0.214, 1.093	0.081^c^	0.390	0.127, 1.199	0.100
Satisfied	0.000	0.000	0.998			
Very satisfied	0.000	0.000	0.998			
Current Occupation (ref. Category I)	1.025	0.844, 1.246	0.803	1.115	0.827, 1.502	0.476
No occupation	1.377	0.165, 11.480	0.768	0.000	0.000	
Category A	‒	‒	‒	‒	‒	‒
Category B	179,497,204.8	0.000	0.999	0.000		
Category C	0.778	0.041	0.867	1.426		
Category D	0.111	0.004	0.213^c^	0.000		
Category E	179,497,204.8	0.000	1.000	‒	‒	‒
Category F	3.556	0.3202	0.386	0.000	0.000	0.999
Category G	‒	‒	‒	‒		
Category H	‒	‒	‒		‒	‒
Pain Scale (Continuous)	1.348	1.117, 1.627	0.002^c^	1.325	1.013, 1.734	0.040^d^
Functional Disability (Continuous)	1.064	0.971, 1.166	0.181^c^	0.991	0.890, 1.103	0.867

Categories A‒I: Brazilian Classification of Occupations; Ref, Reference;

a OR, Odds Ratio;

b aOR, Adjusted Odds Ratio; CI, Confidence Interval.

c p-value < 0.25;

d p-value < 0.05.

### *Prevalence of low back pain at some point in life*

Anthropometric variables such as weight, height, and BMI failed the multicollinearity test, resulting in their exclusion from the model. Sociodemographic factors: spatial distribution, race, marital status, and education were included in the multivariate analysis (*p* < 0.25) (Table 7). The authors observed that widowers had a higher prevalence of LBP at some point in their lives than single individuals (*p* < 0.05) (Table 7). Among the clinical and behavioral characteristics associated with low back pain at some point in life, the following variables were included in the multivariate regression: physical activity, SAH, and Diabetes Mellitus (DM) (*p* < 0.25) (Table 8). The authors observed that the presence of DM was associated with a higher prevalence of low back pain at some point in life (*p* < 0.05) (Table 8). The time of exposure to the previous occupation was excluded from the analysis due to multicollinearity. Previous occupation, satisfaction with the previous occupation, current occupation, satisfaction with the current occupation, and functional disability were included in the multivariate analysis (*p* < 0.25) (Table 9). The authors observed that Occupation, satisfaction with previous occupation, current occupation, and satisfaction with current occupation are related. The authors found that the lower the functional disability, the higher the prevalence of LBP at some point in life (*p* < 0.05) (Table 9).

**Table 7:** Sociodemographic characteristics associated with low back pain at some point in the lives of the 328 participants.

	OR^a^	95% CI^a^	p-value^a^	OR^b^	95% CI^b^	p-value^b^
Spatial distribution (ref. East Zone)	1.238	0.909, 1.685	0.175^c^	1.243	0.909, 1.699	0.172
North	0.617	0.192, 1.989	0.419	0.434	0.116, 1.619	0.214
West	0.790	0.242, 2.582	0.696	0.609	0.162, 2.292	0.463
South	1.300	0.359, 4.712	0.690	0.834	0.199, 3.496	0.804
Race (ref. Black)	1.015	0.821, 1.256	0.888	1.027	0.827, 1.275	0.810
Yellow	1.138	0.112, 11.530	0.913	0.796	0.074, 8.561	0.851
White	1.082	0.560, 2.089	0.815	1.161	0.576, 2.337	0.677
Indigenous	612,766,319.7	0.000	1.000	387,999,751.3	0.000	1.000
Mixed Race	1.571	0.793, 3.114	0.195^c^	1.673	0.809, 3.458	0.165
Marital status (ref. Single)	1.264	1.004, 1.591	0.046^c^	1.248	0.984, 1.584	0.068
Common-law Marriage	2.000	0.181, 22.056	0.571	2.547	0.206, 31.496	0.466
Married	2.054	0.850, 4.962	0.110^c^	2.313	0.905, 5.912	0.080
Divorced	2.476	0.779, 7.869	0.124^c^	2.720	0.817, 9.054	0.103
Widowed	2.833	1.076, 7.458	0.035^c^	3.492	1.219, 10.001	0.020^d^
Education (ref. Postgraduate)	0.865	0.618, 1.210	0.398	0.923	0.650, 1.310	0.654
Illiterate	2.000	0.141, 28.416	0.609	1.342	0.084, 21.391	0.835
No Education	0.250	0.028, 2.237	0.215^c^	0.091	0.008, 1.039	0.054
First Degree	1.302	0.254, 6.680	0.752	0.869	0.147, 5.124	0.877
Second Degree	0.632	0.116, 3.437	0.595	0.499	0.081, 3.084	0.454
Graduation	1.167	0.124, 10.990	0.893	0.741	0.068, 8.033	0.805

Classes A‒E: Economic Classification of Brazil established by the Brazilian Association of Research Companies; Ref, Reference;

a OR, Odds Ratio;

b aOR, Adjusted Odds Ratio; CI, Confidence Interval.

c p-value < 0.25;

d p-value < 0.05.

**Table 8:** Clinical and behavioral characteristics associated with low back pain at some point in the lives of the 328 participants.

	OR^a^	95% CI^a^	p-value^a^	OR^b^	95% CI^b^	p-value^b^
Physical Activity (ref. Sedentary)	1.191	0.892, 1.591	0.236^c^	1.190	0.887, 1.598	0.246
Insufficiently active	1.043	0.458, 2.376	0.919	1.041	0.450, 2.403	0.926
Active	1.486	0.713, 3.096	0.290	1.567	0.746, 3.295	0.236
Very Active	1.623	0.447, 5.894	0.462	1.422	0.385, 5.246	0.598
Systemic Arterial Hypertension	1.438	0.815, 2.537	0.210^c^	1.333	0.747, 2.381	0.330
Diabetes Mellitus	2.086	1.075, 4.049	0.030^c^	1.968	1.005, 3.854	0.048^d^

Ref, Reference;

a OR, Odds Ratio;

b aOR, Adjusted Odds Ratio; CI, Confidence Interval.

c p-value < 0.25;

d p-value < 0.05.

**Table 9:** Occupation and functional disability associated with low back pain at some point in the lives of the 328 participants.

	OR^a^	95% CI^a^	p-value^a^	OR^b^	95% CI^b^	p-value^b^
Previous Occupation (ref. Category I)	0.878	0.724, 1.066	0.188^c^	0.949	0.464, 1.942	0.886
Category A	0.333	0.016, 7.140	0.482	‒	‒	‒
Category B	53,849,161.3	0.000	1.000	0.260	0.000	1.000
Category C	6.667	0.607, 73.195	0.121^c^	0.000	0.000	0.999
Category D	2.000	0.166, 24.069	0.585	‒	‒	‒
Category E	538,491,614.3	0.000	0.999	2.646	0.000	1.000
Category F	0.925	0.241, 3.557	0.910	0.000	0.000	0.999
Category G	1.500	0.200, 11.236	0.693	13.525	0.000	1.000
Category H	0.976	0.231, 4.123	0.976	0.000	0.000	0.999
Satisfaction Previous Occupation (ref. Unsatisfied)	1.601	0.991, 2.584	0.054^c^	1.688	0.429, 6.639	0.454
Satisfied	1.476	0.521, 4.183	0.464	0.178	0.003, 9.803	0.399
Very Satisfied	2.444	0.811, 7.368	0.112^c^	0.350	0.001, 179.870	0.742
Current Occupation (ref. Categoria I)	0.978	0.875, 1.093	0.695	0.978	0.461, 2.073	0.953
No Occupation	2.152	0.587, 7.893	0.248^c^	‒	‒	‒
Category A	‒	‒	‒	‒	‒	‒
Category B	0.667	0.032, 14.033	0.794	0.102	0.000, 35.763	0.445
Category C	4.667	0.404, 53.950	0.217^c^	0.011	0.000	1.000
Category D	0.667	0.032, 14.033	0.794	0.270	0.002, 38.942	0.605
Category E	107,698,322.9	0.000	1.000	‒	‒	‒
Category F	2.476	0.544, 11.272	2.476	‒	‒	‒
Category G	‒	‒	‒	‒	‒	‒
Category H	‒	‒	‒	‒	‒	‒
Satisfaction Current occupation (ref. Unsatisfied)	3.116	0.974, 9.961	0.055^c^	1.717	0.278, 10.617	0.561
Satisfied	0.909	0.074, 11.194	0.941	0.000	0.000	0.999
Very satisfied	5.000	0.302, 82.738	0.261	0.000	0.000	0.999
Functional Disability (Contínuous)	0.943	0.890, 0.999	0.045^c^	0.677	0.475, 0.964	0.030^d^

Categories A‒I, Brazilian Classification of Occupations; Ref, Reference;

a OR, Odds Ratio;

b aOR, Adjusted Odds Ratio; CI, Confidence Interval.

c p-value < 0.25;

d p-value < 0.05.

## Discussion

The estimates revealed a high prevalence of LBP in the periods assessed. The findings are in line with research carried out in other Brazilian cities. In Belém-PA, the prevalence was 56.1 % at the time of the interview and 91.7 % in the last year. The prevalence in Barra Mansa was higher than that of Manaus-AM (42.6) at the time of the interview and similar to the last year (93.7 %)^4^ Regarding the prevalence at some point in life, Barra Mansa had a higher rate than that of São Paulo (62.6 %) and lower than that of Belém (85.3 %)^30^^,^^31^

David et al. (2020)^3^ state that the difference between the states with the highest and lowest prevalence is 32 % and that Rio de Janeiro is the state with the lowest age-standardized rate (12 %) observed between 1990 and 2017. However, they emphasize that the prevalence of LBP increases with age over the years. For this reason, the prevalence of LBP at the time of the interview in Barra Mansa was higher than that of Brazil (25 %)^5^ However, the literature presents an inconsistency in the definitions of LBP prevalence and in the regions investigated, which contributes to the variation observed globally, with rates between 21 % and 75 % in elderly populations^12^

This heterogeneity is also evidenced by systematic reviews that identify high prevalence rates of LBP, especially in low- and middle-income countries. In Africa, the current and annual prevalence of LBP in adults is 39 % and 57 %, respectively, while in Latin America, it ranged from 9 % to 81 %, with up to 67 % in the last year. These data reinforce that, although frequently associated with the elderly population, LBP also affects children, adolescents, and workers, reducing its broad age distribution and relevance as a public health problem in different socioeconomic contexts^32^

Studies indicate that living in urban areas is a protective factor against functional disability. It is possible that elderly people who live in urban areas generally have better living conditions, greater availability, access to preventive services, and specialized medical care^33^ On the other hand, the present study showed that the majority of participants lived in the North region of Barra Mansa (41.0 %) and that the elderly in the West region of Barra Mansa had lower LBP at the time of the interview than those who lived in the East region, an urban area, however, with peripheral neighborhoods. There was no association between spatial distribution and LBP in the last year.

When the variables age, sex, and income were analyzed, none of them showed an association with a higher prevalence of LBP in the last year. Most of the elderly had a mean age of 70.06 ± 6.71 years, a low education level, and a low income. However, a significant association was found between sex and the presence of LBP, with the highest prevalence of LBP occurring in females, contrary to what was found in the study by Sato et al. (2021)^30^

The anatomical and functional differences between sexes affect musculoskeletal health. Factors such as height, muscle mass, and adaptation to physical effort are related to the increased risk of injuries and chronic pain in women. Added to this, the ergonomic loads imposed by domestic activities and work outside the home increase this risk^34^

Higher education and income are directly related to greater and better access to health services, access to information and the viability of a healthier lifestyle, in addition to better working conditions, representing less exposure to occupational risks, as highlighted by other population-based Brazilian studies of population-based^4^^,^^30^ However, the study by Malta et al. (2015)^35^ observed that low education increases the prevalence of functional limitations, corroborating current findings. In addition, the elderly Brazilian population with low education, low income, worse self-rated health, and chronic diseases has high scores of associated functional disability^31^

This scenario is consistent with what is observed globally: Low- and Middle-Income Countries (LMICs) such as India, Africa, and Latin America have the highest rates of disability due to LBP, driven by social determinants, population aging, and excessive use of ineffective institutions. In Brazil, the economic cost of LBP between 2012 and 2016 (social impact) was estimated at US$ 2.2 billion^32^

Still, according to Sharma; McAuley (2022),^32^ the widespread use of ineffective and costly interventions for LBP in LMICs such as Brazil and India contradicts clinical guidelines and has negative impacts on public health. In Brazil, >880.000 imaging tests were performed between 2012 and 2016, while in India, all patients attending orthopedic clinics underwent imaging tests, even without a clear indication. Furthermore, the number and costs of spinal surgeries increased significantly in Brazil between 1995 and 2014. Outdated practices such as bed rest are still recommended by most physicians. These factors are significant for economic and social losses, putting the Sustainable Development Goals at risk recommended by the World Health Organization (WHO).

In this study, white participants presented more LBP at the time of the interview than those of black race/color, contrary to what was found in the study by Sato et al. (2021)^30^ The association between pain and race is not well established. Among the low-income white population, rates of chronic pain resemble those of other ethnic groups in similar socioeconomic situations, despite the black population of our country being more significant in the less favored classes, suggesting that socioeconomic factors are a strong determinant of the experience of pain, regardless of race^30^^,^^36^

On the other hand, a secondary analysis of a prospective cohort study of adults with LBP from seventy-seven primary care clinics in four geographic regions of the United States of America (USA) found that black and Hispanic individuals were more likely to develop chronic LBP than white individuals^37^

This disparity may be partially explained by the implementation of health equity policies by the WHO to ensure accessibility and quality of health services for people with LBP, as non-Hispanic white individuals with chronic LBP are more likely treatments^38^

In contrast, some studies have found no racial/ethnic differences in quality-of-life measures among patients with chronic pain. Racial/ethnic disparities were observed among Hispanics, non-Hispanic Blacks, and non-Hispanic Whites with chronic pain who sought care at a multidisciplinary pain management center and found no differences in pain-related severity or disability^36^ A study of over 5.000 older military veterans found that LBP-related outcomes did not differ by race/ethnicity over 24-months. National cross-sectional surveys have found no association between race/ethnicity and the prevalence of chronic pain in any body region^37^ This reinforces that, in fact, the LBP wall is more associated with socioeconomic factors such as education, income and exposure to risks.

The presence of LBP was significantly associated with lower BMI among participants. Researchers point out that although the relative risks of a high BMI become less pronounced with advancing age, the gradual increase in BMI and obesity among older adults, especially among women, is worrying, similar to what was found in this study (mean BMI 27.26±4.81; 28±4.89 female; 35±4.66 male). The effects of menopause and behavioral factors such as improved ability to prepare food may be related to higher BMI among women^39^

Furthermore, the presence of DM was associated with a higher prevalence of LBP at some point in life. Recent studies indicate that DM is associated with an increased prevalence of chronic LBP among elderly people. The underlying mechanisms include inflammation and musculoskeletal complications in people with DM, which can worsen pain conditions and lead to higher rates of functional disability compared to those without DM^40^

The level of physical activity was associated with LBP. Very active elderly people (moderate physical activities lasting more than twenty minutes three to five days a week) had less LBP at the time of the interview than sedentary elderly people (no physical activity for ten continuous minutes). Studies have evaluated the relationship between physical activity and LBP, highlighting the importance of defining the frequency, duration, intensity, and type of physical activity with the greatest protective effect against the development of LBP, as medium to high levels of physical activity have been shown to be effective in reducing this risk^41^

In this study, the analysis (study design) of the variables’ satisfaction with previous occupation, current occupation, pain scale, and functional capacity showed that greater functional disability is associated with worse LBP at the time of the interview, and a higher pain scale is related to a higher prevalence of LBP in the last year. It was also observed that lower functional disability is associated with a higher prevalence of LBP at some point in life, corroborating other studies^30^^,^^42^

Addressing functional limitations may be essential for pain control, especially when less functional disability is associated with LBP. The satisfaction with occupational roles influences both pain perception and functional disability, highlighting the multifaceted nature of LBP's impact on everyday life and general well-being^42^

In this study, there was a predominance of service workers, such as salespeople in stores and markets, whose main exposure to work was carrying heavy loads. However, other occupations, such as members of the armed forces, police, military firefighters, and science and arts professionals, mainly exposed to standing (64.1 %) and sitting (29.8 %), respectively, were also identified. According to Alzahrani et al. (2019),^41^ the participation in occupational activities, including frequent lifting of loads and physically demanding work, was considered a medium to strong risk factor for LBP. The studies, therefore, must consider both occupational and non-occupational physical activities in order to reflect people's daily exposure to all types of activities.

The results found on occupation and functionality associated with LBP stand out from studies already carried out in other Brazilian cities and deserve attention. It is proposed that occupational activities and exposure time in previous and current occupations may significantly influence the prevalence of LBP and functional disability.

When asked about how physical health or emotional problems interfere with social activities, the category slightly, moderately, or extremely shows more association than not at all, similar to the elderly population studied in Manaus and Belém^4^^,^^30^ The emotional health of the elderly is closely related to their functional capabilities, and regional differences in Brazil can affect this relationship. In the Northeast, the elderly tend to have a more robust support network and spend more time with their peers, which can help reduce loneliness, the prevalence of depression, and the negative perception of their health. On the other hand, in the Southeast, factors such as financial restrictions, subjective poverty, and lack of meeting basic needs can contribute to the emergence of functional limitations. These factors hinder the social participation of elderly people, especially among the oldest ones^43^

Therefore, successful aging involves more than the absence of disease, including the preservation of physical, mental, and social capacities. Highlight that regular physical activity, combined with social and cognitive engagement, contributes to the prevention of chronic diseases and to the reduction of the risk of cognitive decline and depression. This combination favors the autonomy and well-being of the elderly, reinforcing the importance of public policies that promote active aging^44^

### *Limitations*

The study, with a non-probabilistic sample and a female majority, highlights limitations in the representativeness and generalization of the results. The lack of regional epidemiological data in Barra Mansa makes accurate calculations difficult, but it highlights the importance of a database to guide public health policies. Furthermore, recall biases related to lifetime low back pain may inflate prevalence estimates, as participants may have difficulty accurately recording past episodes of pain, leading to exaggerated or inaccurate reports. The selection may also be a relevant factor, as participation was more likely among healthier elderly, or may have influenced the results obtained by underestimating the prevalence of conditions in more vulnerable populations. The authors acknowledge the specific limitations of the cross-sectional data used in this study, which restrict the possibility of establishing causal relationships. Since this work is exploratory in nature, emphasis was placed on the initial descriptive analysis of the data. More robust methodological approaches, such as propensity score matching or sensitivity analyses, were not applied at this time, but are planned for future stages of the research, preferably with longitudinal designs that allow for more in-depth monitoring of the variables investigated.

### *Clinical implications*

The LBP in pain in occupationally active older adults can compromise productivity, independence, and quality of life, and increase the risk of isolation and functional decline. Effective management requires specific guidelines for the treatment of low back pain, such as group exercise programs for elderly women (stretching, muscle strengthening, and abdominal stability), walking, water activities, and health education programs focusing on ergonomics and self-care^45^ When innovative at the community level, these strategies not only improve functionality but also prevent disability, decrease hospitalizations, and reduce treatment costs, supporting the development of more effective and cost-effective clinical guidelines.

## Conclusions

This study reveals a strikingly high prevalence of LBP among elderly Brazilians, with over 90 % experiencing pain in the past year. The multifactorial nature of LBP ‒ spanning sociodemographic (sex, race), clinical (hypertension, diabetes), and behavioral (physical activity) domains ‒ highlights the need for integrated, lifespan-focused interventions. Notably, modifiable factors like physical inactivity and functional disability offer actionable targets for public health strategies, particularly in LMICs facing similar aging-related challenges. By aligning with WHO initiatives on active aging, these findings advocate for community-based programs to reduce disability and economic burdens. Future longitudinal studies should validate causal relationships and optimize interventions for diverse elderly populations.

## Declaration of generative AI and AI-assisted technologies in the writing process

During the preparation of this work, the author(s) used [ChatGPT] was used only to improve language and readability. After using this tool/service, the author(s) reviewed and edited the content as needed and take(s) full responsibility for the content of the publication.

## Definitions

Declare that the term sex refers to a set of biological attributes that are associated with physical and physical characteristics.

## Institutional review board statement

The study was conducted according to the guidelines of the Declaration of Helsinki and approved by the Research Ethics Committee of the Faculty of Medicine of the University of São Paulo, São Paulo, Brazil (protocol code: 189/16, date of approval: 8 June 2016).

## Informed consent statement

Informed consent was obtained from all participants involved in the study.

## Submission declaration

This article has not been published previously (except in the form of an abstract or academic thesis), it is not under consideration for publication elsewhere. Its publication was approved by all authors and tacitly or explicitly by the responsible authorities where the work was carried out, and that if accepted, it will not be published elsewhere in the same form, in English or in any other language, including electronically, without the written consent of the copyright-holder.

## Declaration of competing interest

The authors declare no conflicts of interest
